# Luteolin reduces necroptosis in the diabetic heart after cardiac arrest and resuscitation by activating sirtuin 3

**DOI:** 10.3389/fnut.2025.1626020

**Published:** 2025-06-25

**Authors:** Jin-Ting Yang, Chun-Yan Jiang, Zi-Yan Zhang, Li-Hui Tang, Yang-Yun Lou, Ling-Bo Qian

**Affiliations:** ^1^Department of Anesthesiology, The Second Affiliated Hospital of Zhejiang University School of Medicine, Hangzhou, China; ^2^School of Basic Medical Sciences and Forensic Medicine, Hangzhou Medical College, Hangzhou, China

**Keywords:** diabetes, post-resuscitation cardiac injury, sirtuin 3, necroptosis, mitochondria, oxidative stress, inflammation, luteolin

## Abstract

**Backgrounds:**

Myocardial impairment resulting from cardiopulmonary resuscitation (CPR) contributes to the elevated mortality in diabetes. Luteolin, a naturally occurring polyphenolic compound abundant in vegetables, fruits, and nuts, has been shown to mitigate myocardial I/R injury in diabetes by suppressing oxidative stress. However, whether luteolin confers cardioprotection following cardiac arrest (CA) and CPR in diabetes remains unclear. Sirtuin 3 (Sirt3), a mitochondrial deacetylase, has been reported to attenuate diabetic cardiomyopathy by reducing oxidative stress and inflammation-mediated necroptosis. Recent evidence suggests that luteolin can upregulate Sirt3 and enhance mitochondrial function. Thus, we hypothesized that luteolin may alleviate post-CA/CPR myocardial injury in diabetes by inhibiting necroptosis through activation of the Sirt3 signaling pathway.

**Methods:**

Diabetes was induced in male Sprague-Dawley rats via a single intraperitoneal injection of streptozotocin (65 mg/kg). Rats were then treated with luteolin (100 mg/kg, i.g.) or Sirt3 inhibitor 3-TYP for 2 weeks. Subsequently, diabetic rats were subjected to 5 min of asphyxia-induced CA followed by CPR. After 6 h of resuscitation, left ventricular function, myocardial infarction, oxidative stress markers, inflammatory cytokine release, mitochondrial function, necroptosis-associated protein expression, and both Sirt3 expression and enzymatic activity were assessed.

**Results:**

Luteolin significantly improved post-resuscitation cardiac function and reduced myocardial infarction, oxidative stress, and pro-inflammatory cytokine levels in diabetic rats. It also inhibited cytosolic Ca^2+^ overload, mitochondrial permeability transition pore opening, and loss of mitochondrial membrane potential. Additionally, luteolin activated Sirt3 and superoxide dismutase 2. Importantly, luteolin increased the expression of Sirt3 and suppressed necroptosis by down-regulating phosphorylated receptor-interacting protein kinase 3 and phosphorylated mixed lineage kinase domain-like protein. The cardioprotective effects of luteolin were abrogated by co-administration of 3-TYP, indicating a critical role for Sirt3 in mediating these benefits.

**Conclusion:**

Luteolin protects diabetic hearts after CA/CPR by suppressing necroptosis, primarily through activation of Sirt3, which dampens oxidative stress and inflammation, and maintains mitochondrial integrity.

## 1 Introduction

Diabetes mellitus affects a growing portion of the global population. According to the International Diabetes Federation, approximately 537 million adults worldwide (1 in 10 adults) had diabetes in 2021, a figure projected to rise to 783 million by 2030.^1^ Patients with diabetes are at significantly increased risk of cardiovascular complications such as myocardial infarction, stroke, and heart failure, which are leading causes of morbidity and mortality in this population (1–3). Despite notable advancements in cardiopulmonary resuscitation (CPR) techniques, cardiac arrest (CA) remains a critical public health concern with poor prognoses, contributing to approximately 15%–20% of global mortality (4, 5). Diabetes is an established risk factor for CA due to sustained hyperglycemia-induced vascular endothelial dysfunction, microangiopathy, and cardiac autonomic neuropathy (6–8). Achieving return spontaneous circulation (ROSC) by timely initiation of CPR is critical for saving patients with CA. However, diabetic patients who achieve ROSC tend to have lower survival rates than non-diabetic individuals (9, 10). A key contributor to this disparity is myocardial injury caused by global ischemia/reperfusion (I/R) insult during resuscitation (8, 11), highlighting the urgent need for effective cardioprotective strategies in this context.

Hyperglycemia exacerbates oxidative stress and inflammatory responses, increasing myocardial vulnerability to I/R injury and diminishing the effectiveness of cardioprotective interventions, which may be responsible for the poor outcome in diabetic patients after CA/CRP (6, 8, 12). Reperfusion following CPR generates an abundance of reactive oxygen species (ROS), which further aggravates oxidative damage and inflammatory cascades. These effects contribute to ongoing cardiomyocyte death and post-resuscitation cardiac dysfunction in diabetes (6, 8, 12). Recent evidence indicates that necroptosis, a form of regulated cell death involving the formation of a protein complex known as the necrosome, responds to oxidative stress and inflammation and plays a critical role in myocardial I/R injury (13–15). Under such stress conditions, receptor-interacting protein kinase 1 (RIPK1) interacts with RIPK3 to form the necrosome, leading to the phosphorylation and activation of RIPK3 (16). Mixed lineage kinase domain-like protein (MLKL), a key executor of necroptosis, is recruited and phosphorylated by the activated RIPK3, which allows its translocation to the cell or organelle membrane to form lethal pores, leading to the release of intracellular contents including highly inflammatory molecules and free radicals, ultimately amplifying cell death (17, 18). Inhibiting necroptosis has been reported to mitigate cardiac I/R injury by reducing oxidative stress and inflammation (17, 19). However, the role of necroptosis in post-resuscitation injury in diabetic hearts remains underexplored.

Sirtuin 3 (Sirt3), a mitochondrial class III histone deacetylase, is essential for maintaining mitochondrial integrity, metabolic balance, redox homeostasis, and cell death under physiological and pathological conditions (20–23). Up-regulation of Sirt3 has been shown to preserve mitochondrial function and ameliorate both diabetic cardiomyopathy (22, 24) and myocardial I/R injury (21, 25, 26). Sirt3 protects the heart at least in part by activating superoxide dismutase 2 (SOD2), a mitochondrial antioxidant enzyme, thereby reducing oxidative stress and necroptosis (24, 27). Additionally, recent studies suggest that Sirt3 can improve myocardial function and survival following CA/CPR (28). These findings point to a potential therapeutic role for Sirt3-mediated necroptosis inhibition in protecting the diabetic heart after resuscitation.

We previously demonstrated that luteolin (3′,4′,5,7-tetrahydroxyflavone), a naturally occurring flavonoid abundant in various vegetables, fruits, and nuts, alleviates myocardial I/R injury in diabetes through its antioxidant actions (11, 29). Notably, luteolin enhances mitochondrial function and reduces ROS accumulation by up-regulating Sirt3 in models of cerebral I/R injury and ultraviolet radiation B-induced skin photoaging (30, 31). Moreover, luteolin has been shown to suppress necroptosis in glucocorticoid-induced osteonecrosis of the femoral head (32). These findings raise the possibility that luteolin could protect the diabetic heart against post-resuscitation I/R injury by modulating necroptosis via Sirt3.

Therefore, the present study was carried out to investigate whether luteolin alleviates myocardial I/R injury following CA/CPR in diabetes, specifically through Sirt3-mediated inhibition of necroptosis.

## 2 Materials and methods

### 2.1 Chemicals

Luteolin was from Tokyo Chemical Industry (Tokyo, Japan). Streptozotocin (STZ), methylthiazolyldiphenyl-tetrazolium bromide (MTT), collagenase, and dimethyl sulfoxide (DMSO) were purchased from Sigma-Aldrich (Saint Louis, MO, USA). The specific Sirt3 inhibitor, 3-(1H-1,2,3-triazol-4-yl)pyridine (3-TYP), was purchased from AbMole BioScience (Houston, TX, USA). Antibodies against Sirt3, RIPK3, and GAPDH were from Cell Signaling Technology (Danvers, MA, USA). Anti-phosphor-RIPK3 (p-RIPK3) was from Abcam (Cambridge, UK). Antibodies against p-MLKL and MLKL, Trizol^®^ Reagents, and myeloperoxidase (MPO) activity assay kit were purchased from Thermo Fisher Scientific (Waltham, MA, USA). The Protein Carbonyl Colorimetric Assay Kit was from Cayman Chemical (Ann Arbor, MI, USA). The mitochondrial membrane potential (ΔΨm) indicator JC-1, mitochondrial permeability transition pore (mPTP) opening indicator calcein AM, mitochondrial superoxide indicator MitoSOX Red, intracellular ROS indicator 2,7-dichlorofluorescin diacetate (DCFDA), cell-permeable Ca^2+^ dye Fura-2 AM, tissue mitochondria isolation kit, ELISA kits for interleukin-1β (IL-1β) and tumor necrosis factor-α (TNF-α), kits for the assay of malondialdehyde (MDA), ATP, and SOD2 activity were all purchased from Beyotime Biotech (Shanghai, China). ELISA kits for 8-hydroxy-2′-deoxyguanosine (8-OHdG), cardiac troponin I (cTnI), and creatine kinase-MB (CK-MB) were from Elabscience (Wuhan, China). Assay kits for mitochondrial respiratory chain complexes I–III were obtained from Solarbio Science & Technology (Beijing, China). Isoflurane was provided by RWD Life Science (Shenzhen, China). Luteolin was dissolved in 0.5% (w/v) sodium carboxymethyl cellulose (CMC-Na). Other chemicals used were of analytical grade.

### 2.2 Induction of diabetes in rats

Male Sprague-Dawley rats (280 ± 20 g), purchased from Shanghai Laboratory Animal Center, were housed under control conditions (22 ± 1°C; 12-h light/dark cycle) with free access to water and standard pellet chow. All animal procedures conformed to the Guide for the Care and Use of Laboratory Animals and were approved by the Ethics Committee for the Use of Experimental Animals in Hangzhou Medical College. Diabetes was induced by a single intraperitoneal injection of STZ (65 mg/kg) following our previous protocol (11). After 72 h, rats with blood glucose levels > 16.7 mM were considered diabetic.

### 2.3 Experimental protocol

Rats were randomly divided into four groups (*n* = 6 per group): (1) Non-diabetic group (ND); (2) Diabetic group (D); (3) Luteolin-treated diabetic group (D+Lut); and (4) 3-TYP and luteolin-treated diabetic group (D+3-TYP+Lut). Luteolin (100 mg/kg) was intragastrically administered for 2 weeks (11). 3-TYP (50 mg/kg) was intraperitoneally injected every other day before luteolin treatment (28, 33). A parallel experiment was conducted to determine the effect of Sirt3 on luteolin-mediated modulation of intracellular Ca^2+^ overload and mPTP opening in ventricular myocytes. After 2 weeks of diabetes, rats underwent the asphyxia-induced CA/CPR procedure.

### 2.4 Asphyxia-induced CA/CPR model

The CA/CPR model was established with slight modifications from the prior protocol (34). Briefly, rats were anesthetized with isoflurane (4% induction, 2% maintenance), intubated with a 14G cannula, and connected to a ventilator. After 5 min of equilibration, vecuronium (2 mg/kg, i.v.) was administered and mechanical ventilation was halted to initiate CA. After 5 min of CA, resuscitation was performed using 100% oxygen ventilation, chest compressions (about 200 compressions/min), and epinephrine (0.02 mg/kg, i.v.). ROSC was confirmed by a sustained mean arterial pressure > 60 mmHg for at least 10 min. Six hours post-ROSC, echocardiography was performed. Heart and serum samples were collected and stored at −80°C for further analysis. Ventricular myocytes and mitochondria were isolated to assess mPTP opening, ΔΨm, and activities of Sirt3 and mitochondrial respiratory chain complexes.

### 2.5 Echocardiography

Rats were anesthetized with 2% isoflurane and cardiac function and morphology were assessed using the Vevo 2100 high-resolution imaging system equipped with a 20 MHz probe (VisualSonics, Toronto, Canada), following the protocol we previously described (35). Left ventricular ejection fraction (EF) and fractional shortening (FS) were calculated to evaluate systolic function.

### 2.6 Assessment of serum biomarkers

After performing echocardiography at the end of 6 h-resuscitation, rat blood was collected and the clear serum supernatant was separated through centrifugation at 3,000 rpm/min at 4°C for 15 min. Serum levels of cTnI and CK-MB, IL-1β and TNF-α after resuscitation were quantified using commercial ELISA kits, according to the manufacturer’s protocols (21, 36).

### 2.7 Determination of myocardial infarction and oxidative stress

After 6 h of resuscitation, myocardial infarction was assessed by the reduction of formazan content. In brief, the heart tissue was incubated with MTT (3 mM) at 37°C for 30 min to form formazan, then homogenized in DMSO (40 ml/g) and centrifuged at 1,000 *g* for 10 min. The absorbance of the supernatant was measured using a microplate reader at 550 nm (11). Cardiac MDA content was analyzed spectrophotometrically following the commercial kit manuals (37). Cardiac 8-OHdG (38, 39) and protein carbonyl levels (40) were measured using ELISA and colorimetric assay kits, respectively, according to the manufacturer’s instructions.

### 2.8 Measurement of MPO and SOD2 activities, and ATP content

Cardiac MPO activity was determined by measuring hydrogen peroxide-mediated oxidation of 3,3′,5,5′-tetramethylbenzidine as previously described (41). Briefly, after 6 h of resuscitation, heart tissue was homogenized and centrifuged at 40,000 *g* for 15 min in a PBS buffer. MPO activity of the supernatant was assayed following the manufacturer’s instructions. SOD2 activity (42) and ATP levels (43) in the heart tissue were quantified using specific commercial kits, following the manufacturer’s instructions.

### 2.9 Measurement of activities of Sirt3 and mitochondrial respiratory chain complexes

After 6 h of resuscitation, ventricular myocardial mitochondria were isolated by differential centrifugation using a commercial kit according to the manufacturer’s protocol (28). The deacetylase activity of Sirt3 in resuspended mitochondrial pellets was measured using a Sirt3 fluorogenic assay kit. with fluorescent intensity recorded at 350 nm excitation and 460 nm emission (28). The activities of mitochondrial respiratory chain complexes I–III were quantified using a microplate reader, strictly adhering to the protocols provided with the commercial kits (33, 44).

### 2.10 Assessment of cytosolic free Ca^2+^, mPTP opening, and ΔΨm

After 6 h of resuscitation, single ventricular myocytes were enzymatically isolated as described previously (11, 45). The myocytes were stabilized in Tyrode’s solution before measurements. As previously reported (46, 47), cytosolic free Ca^2+^ levels were detected using Fura-2 AM, with fluorescence at 340/380 nm excitation and 510 nm emission. Ca^2+^ concentration was calculated as follows: [Ca^2+^]_*i*_ = K_*d*_ × [(R-R_*EGTA*_)/(R_*Triton–X*__100_–R)] × (F_380*EGTA*_/F_380*Triton–X*__100_) (K_*d*_ = 225 nmol/L, *R* = F_340_/F_380_). The opening of mPTP was detected by calcein fluorescence quenching at 488 nm excitation and 505 nm emission, and ΔΨm was evaluated by JC-1 aggregate fluorescence at 488 nm excitation and 590 nm emission in myocytes using a microplate reader, following our previous studies (11, 45) and others (48).

### 2.11 Determination of ROS and mitochondrial superoxide

After 6 h of resuscitation, intracellular ROS and mitochondrial superoxide generation in isolated cardiomyocytes were evaluated using DCFDA and mitoSOX Red, respectively, measured by a fluorescence plate reader at 488 nm excitation and 535 nm emission (DCFDA) and 510 nm excitation and 580 nm emission (mitoSOX Red), following the manufacturer’s instructions (49, 50).

### 2.12 Reverse transcription and quantitative polymerase chain reaction (qRT-PCR)

After 6 h of resuscitation, total RNA was extracted from heart tissue using Trizol^®^ Reagent. qRT-PCR was performed as previously reported (51) using the following primers: TNF-α (F): GAAACACACGAGACGCTGAA, TNF-α (R): CAGTCTGGGAAGCTCTGAGG; IL-1β (F): CAGCAGCAT CTCGACAAGAG, IL-1β (R): CATCATCCCACGAGTCACAG.

### 2.13 Western blotting

After 6 h of resuscitation, protein expression levels of Sirt3, RIPK3, p-RIPK3, MLKL, and p-MLKL in heart tissues were determined by western blotting as we previously described (11, 35).

### 2.14 Statistical analysis

Data are expressed as mean ± SD. Statistical comparisons among groups were performed using one-way ANOVA followed by Tukey’s *post-hoc* test (GraphPad Prism 9.5, San Diego, CA, USA). A value of *p* < 0.05 was considered statistically significant.

## 3 Results

### 3.1 Blood glucose levels in all groups

**FIGURE 1:**
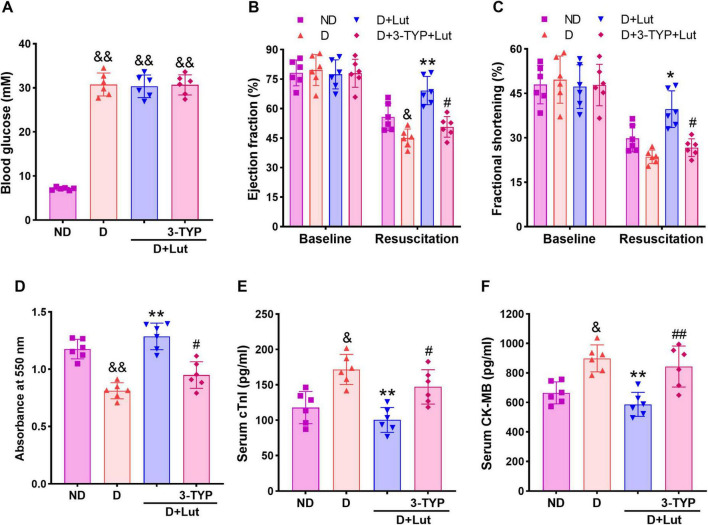
Effects of luteolin on attenuating cardiac injury in diabetic rats after cardiac arrest for 5 min and cardiopulmonary resuscitation (CA/CPR). **(A)** Effect of luteolin on blood glucose in diabetic rats. **(B)** The left ventricular ejection fraction (EF) and **(C)** fractional shortening (FS) in all groups after 6 h of resuscitation. **(D)** The myocardial infarction indicated by formazan content and myocardial injury markers **(E)** serum cTnI and **(F)** CK-MB in all groups after 6 h of resuscitation. ND, non-diabetic rats; D, diabetic rats; D+Lut, diabetic rats treated with luteolin (100 mg/kg, i.g.); D+3-TYP+Lut, diabetic rats treated with Sirt3 inhibitor 3-TYP (50 mg/kg, i.p.) and luteolin. Luteolin and 3-TYP were administered for 2 weeks after the establishment of diabetes. Baseline, before CA; Resuscitation, after 6 h of resuscitation. All data are presented as mean ± SD; *n* = 6 rats per group; ^&^*p* < 0.05, ^&⁣&^*p* < 0.01 versus ND; *p < 0.05, **p < 0.01 versus D; ^#^*p* < 0.05, ^##^*p* < 0.01 versus D+Lut.

The effect of luteolin on blood glucose levels were evaluated. After 2 weeks, blood glucose levels in the D group remained elevated (> 25 mM), significantly higher than those in the ND group (*p* < 0.01). Treatment with luteolin alone or in combination with 3-TYP did not significantly alter hyperglycemia in diabetic rats (Figure 1A), consistent with our previous findings (11, 35). These results suggest that the cardioprotection of luteolin on diabetic rats after CA/CPR is independent of reducing blood glucose.

### 3.2 Luteolin attenuated cardiac injury in diabetic rats after CA/CPR

Echocardiography was performed in rats to assess the improving effect of luteolin on post-resuscitation cardiac function. As shown in Figures 1B, C, baseline values of left ventricular EF and FS were not significantly different among groups. Following CA/CPR, both EF and FS were significantly decreased in diabetic hearts compared to the ND group (*p* < 0.05). Luteolin treatment significantly improved systolic function (*p* < 0.01 versus D), whereas co-administration of the Sirt3 inhibitor 3-TYP attenuated these beneficial effects (*p* < 0.05 versus D+Lut group). Heart tissue viability, assessed via formazan content, was determined to indicate the myocardial infarction. As shown in Figure 1D, myocardial viability was significantly reduced in the D group (*p* < 0.05 versus ND). Serum levels of cardiac injury biomarkers cTnI and CK-MB were markedly elevated following CA/CPR in diabetic rats compared to the ND group (*p* < 0.01; Figures 1E, F). Luteolin significantly mitigated these cardiac impairments (*p* < 0.01 versus D), which were markedly suppressed by 3-TYP (*p* < 0.05 versus D+Lut). These findings indicate that luteolin improves cardiac recovery after resuscitation, potentially through modulation of Sirt3-dependent pathways.

### 3.3 Luteolin reduced oxidative stress and inflammation in diabetic rats after CA/CPR

**FIGURE 2:**
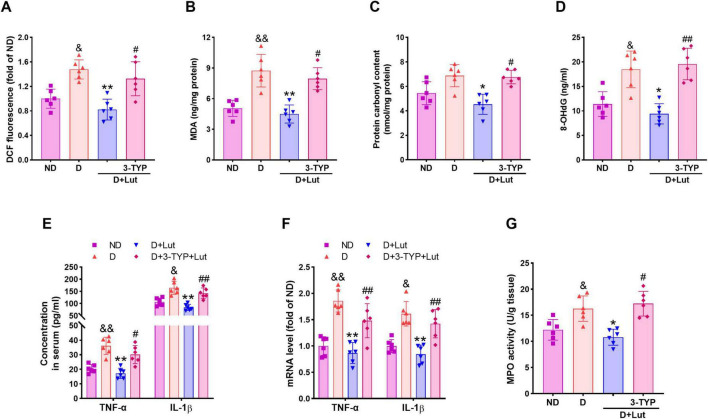
Effects of luteolin on reducing oxidative stress and inflammatory responses in diabetic rats after CA/CPR. **(A)** The cardiac ROS level indicated by DCF fluorescence, **(B)** MDA, **(C)** protein carbonyl, and **(D)** 8-OHdG in all groups after 6 h of resuscitation. **(E)** Serum and **(F)** cardiac inflammatory cytokines TNF-α and IL-1β, and **(G)** cardiac MPO activity in all groups after 6 h of resuscitation. Rats were divided into 4 groups as shown in the above figure. All data are presented as mean ± SD; *n* = 6 rats per group; ^&^*p* < 0.05, ^&⁣&^*p* < 0.01 versus ND; *p < 0.05, **p < 0.01 versus D; ^#^*p* < 0.05, ^##^*p* < 0.01 versus D+Lut.

To assess the attenuating effects of luteolin on oxidative stress and inflammation in diabetic rats after CA/CPR, ROS production, oxidative damage markers, and inflammatory cytokines were measured. As shown in Figure 2A, CA/CPR significantly increased cardiac ROS production in diabetic rats (*p* < 0.05 versus ND), which was markedly attenuated by luteolin treatment (*p* < 0.01 versus D). The antioxidant effect of luteolin was significantly reduced by 3-TYP (*p* < 0.05). Oxidative damage markers, MDA and 8-OHdG, were significantly elevated in the D group (*p* < 0.05 versus ND, Figures 2B, D), and protein carbonyl levels showed a moderate, non-significant increase (Figure 2C). Luteolin treatment effectively reduced these markers (*p* < 0.05), but its effect was reversed by 3-TYP (*p* < 0.05). Inflammatory cytokines TNF-α and IL-1β were significantly elevated in both serum (Figure 2E) and heart tissue (Figure 2F) of diabetic rats after CA/PCR (*p* < 0.05 versus ND), along with increased cardiac MPO activity (Figure 2G). These proinflammatory responses were markedly reduced by luteolin (*p* < 0.05 versus D), and this reduction was counteracted by 3-TYPP (*p* < 0.05 versus D+Lut). These results suggest that luteolin reduces oxidative stress and inflammation in diabetic rats after CA/CPR, possibly via activating Sirt3.

### 3.4 Luteolin reduced mitochondrial superoxide and up-regulated Sirt3 in diabetic hearts after CA/CPR

**FIGURE 3:**
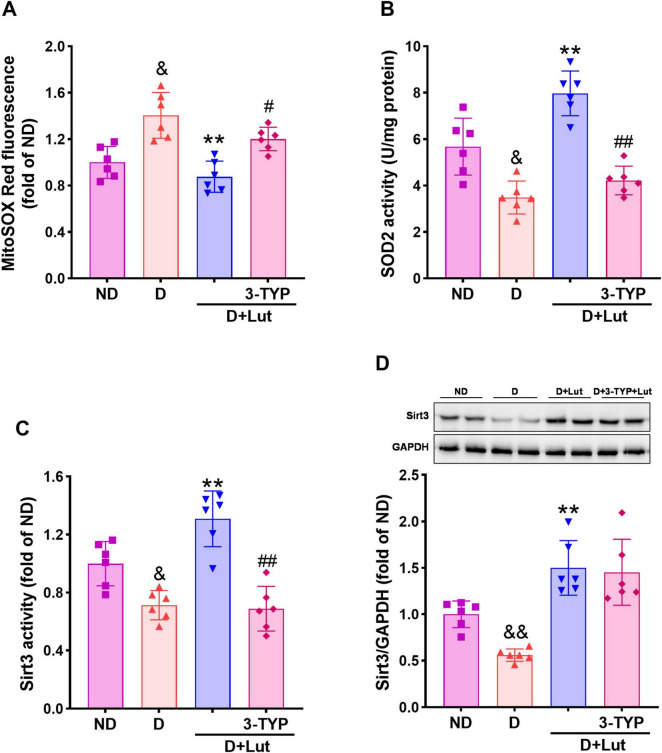
Effects of luteolin on reducing mitochondrial oxidative stress in diabetic rat hearts after CA/CPR. **(A)** The mitochondrial superoxide level indicated by MitoSox Red fluorescence, **(B)** SOD2 activity, **(C)** Sirt3 activity, and **(D)** protein expression in all rat hearts after 6 h of resuscitation. Rats were divided into 4 groups as shown in the above figure. All data are presented as mean ± SD; *n* = 6 rats per group; ^&^*p* < 0.05, ^&⁣&^*p* < 0.01 versus ND; **p < 0.01 versus D; ^#^*p* < 0.05, ^##^*p* < 0.01 versus D+Lut.

To evaluate luteolin’s attenuation of myocardial mitochondrial oxidative stress in diabetic rats following CA/CPR, we measured mitochondrial superoxide levels, SOD2 activity, and Sirt3 activity/expression. Mitochondrial superoxide levels, indicated by MitoSOX Red fluorescence, were significantly elevated in diabetic rat hearts following CA/CPR (*p* < 0.05 versus ND; Figure 3A). Luteolin significantly reduced mitochondrial superoxide (*p* < 0.01 versus D), an effect weakened by 3-TYP (*p* < 0.05). As shown in Figure 3B, SOD2 activity was decreased in diabetic hearts after resuscitation, but this was significantly reversed by luteolin (*p* < 0.01). This improving effect of luteolin was counteracted by 3-TYP (*p* < 0.01). Sirt3 activity and protein expression were markedly reduced in diabetic hearts after resuscitation (*p* < 0.05 versus ND), which was significantly reversed by luteolin (*p* < 0.01, Figures 3C, D). 3-TYP abrogated the improving effect of luteolin on Sirt3 activity (*p* < 0.01) but not the protein expression. These results demonstrate that luteolin reduces mitochondrial oxidative stress in diabetic rat hearts following CA/CPR, potentially through activation of the Sirt3-SOD2 pathway.

### 3.5 Luteolin reduced mitochondrial impairments in diabetic hearts after CA/CPR

**FIGURE 4:**
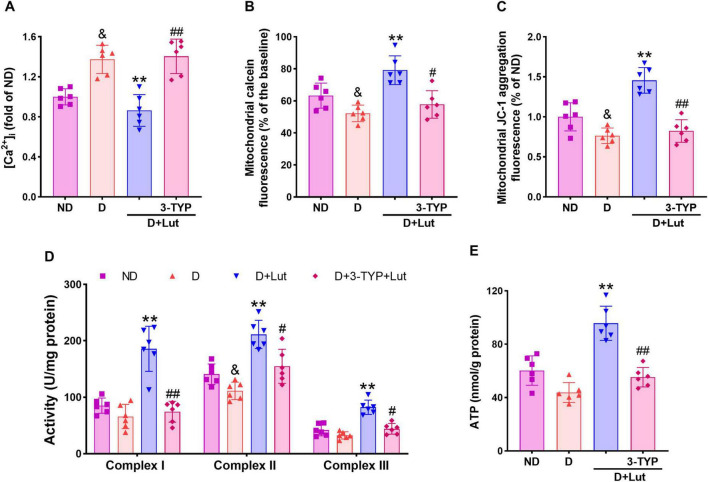
Effects of luteolin on attenuating mitochondrial impairments in diabetic rat hearts after CA/CPR. **(A)** The free intracellular Ca^2+^ level indicated by cell-permeable Ca^2+^ probe Fura-2 AM, **(B)** mPTP opening indicated by the reduction of calcein fluorescence, **(C)** mitochondrial membrane potential (ΔΨm) indicated by JC-1 aggregation fluorescence, **(D)** activities of mitochondrial respiratory chain complexes I–III, and **(E)** ATP production in all rat hearts after 6 h of resuscitation. Rats were divided into 4 groups as shown in the above figure. All data are presented as mean ± SD; *n* = 6 rats per group; ^&^*p* < 0.05 versus ND; **p < 0.01 versus D; ^#^*p* < 0.05, ^##^*p* < 0.01 versus D+Lut.

To assess luteolin’s protective effects against myocardial mitochondrial dysfunction in diabetic rats following CA/CPR, we measured cytosolic Ca^2+^ levels, mPTP opening, ΔΨm, activities of respiratory chain complexes I–III and ATP production. Following CA/CPR, diabetic rats exhibited significant mitochondrial dysfunction, as evidenced by elevated cytosolic Ca^2+^ (Figure 4A), increased mPTP opening (Figure 4B), and decreased ΔΨm (Figure 4C) (*p* < 0.05 versus ND). These impairments were significantly ameliorated by luteolin (*p* < 0.01 versus D), with 3-TYP reversing the improvements (*p* < 0.05). Luteolin also reversed the reduced activity of mitochondrial respiratory chain complexes I–III and ATP production (*p* < 0.01 versus D), both of which were significantly inhibited by 3-TYP (*p* < 0.05, Figures 4D, E). Our findings demonstrate that luteolin ameliorates CA/CPR-induced mitochondrial dysfunction in diabetic hearts, likely through Sirt3-mediated preservation of mitochondrial integrity.

### 3.6 Luteolin suppressed necroptosis in diabetic hearts after CA/CPR

**FIGURE 5:**
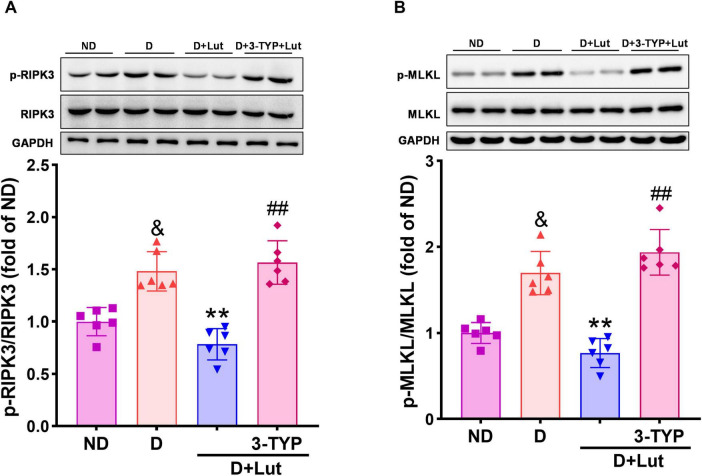
Effects of luteolin on down-regulating necroptosis-related factors in diabetic rat hearts after CA/CPR. The protein expression of **(A)** p-RIPK3 and **(B)** p-MLKL in all rat hearts after 6 h of resuscitation. Rats were divided into 4 groups as shown in the above figure. All data are presented as mean ± SD; *n* = 6 rats per group; ^&^*p* < 0.05 versus ND; **p < 0.01 versus D; ^##^*p* < 0.01 versus D+Lut.

To investigate luteolin’s inhibition of necroptosis in diabetic hearts after CA/CPR, we analyzed the activation status of necroptotic executioners through immunoblotting of p-RIPK3 and p-MLKL. As shown in Figure 5, CA/CPR significantly increased levels of necroptosis-related proteins p-RIPK3 and p-MLKL in diabetic hearts (*p* < 0.01 versus ND), which were significantly reversed by luteolin (*p* < 0.01). Co-treatment with 3-TYP abrogated the inhibitory effects of luteolin on necroptotic factors (*p* < 0.01 versus D+Lut), indicating that luteolin-mediated suppression of necroptosis is dependent on Sirt3 activation. These data suggest that luteolin suppresses CA/CPR-induced activation of necroptotic executioners (p-RIPK3 and p-MLKL) in diabetic hearts, potentially through Sirt3-mediated signaling pathways.

## 4 Discussion

This study demonstrated that: (1) Sirt3 mediates the cardioprotective effects of luteolin against post-resuscitation injury in diabetes; (2) Reduction of oxidative stress and inflammation via the Sirt3 signaling pathway contributes to preservation of mitochondrial function; and (3) Luteolin-activated Sirt3 signaling suppresses necroptosis, leading to improved cardiac outcomes after CA/CPR in diabetic rats. In agreement with our previous studies (11, 35), luteolin treatment did not significantly alter blood glucose levels, indicating that its cardioprotective effects are independent of glycemic control. These findings support the hypothesis that luteolin (100 mg/kg) protects the diabetic heart from post-resuscitation injury primarily by activating Sirt3, which mitigates oxidative stress, inflammation, and mitochondrial dysfunction, ultimately inhibiting necroptosis.

Diabetic patients experience worsened outcomes after CA/CPR due to multifactorial pathophysiology, including heightened oxidative stress, inflammation, and mitochondrial dysfunction (8). CA and subsequent CPR cause global myocardial I/R injury, which is exacerbated in diabetes due to chronic hyperglycemia. Elevated ROS levels induced by high glucose in the diabetic myocardium oxidize lipids, proteins, and DNA, promoting tissue damage and cardiomyocyte death (19). Consistent with our previous reports (11, 45), the current study shows that luteolin significantly reduces myocardial infarction, lowers serum markers of cardiac injury (cTnI and CK-MB), and improves systolic function following CA/CPR in diabetic rats. These protective effects are accompanied by marked suppression of myocardial ROS production and reduced accumulation of oxidized lipids, DNA, and proteins, indicating that luteolin’s antioxidant properties contribute to myocardial protection. Oxidative stress and inflammation are mutually reinforcing in diabetic cardiomyopathy and I/R injury (8, 12, 24). Mitochondria are both the primary source and major target of ROS, and mitochondrial ROS production fuels inflammatory cascades via cytokine release and immune cell infiltration (8, 12). We recently revealed that luteolin inhibits the infiltration of inflammatory cells and cytokines in the diabetic rat heart and alleviates diabetic cardiomyopathy (36). The present study indicates that luteolin reduces mitochondrial superoxide production and suppresses inflammatory markers such as TNF-α, IL-1β, and MPO in diabetic hearts after resuscitation. These results support a mechanism whereby luteolin interrupts the feed-forward cycle between mitochondrial ROS and inflammation, conferring protection against post-resuscitation injury. Necroptosis connecting oxidative stress and inflammation has been identified in infarcted myocardium, indicating its adverse role in heart failure after I/R (19). Although necroptosis contributes to diabetic cardiomyopathy and cardiac I/R injury, the role and prevention of necroptosis in diabetic heart injury after resuscitation remain elusive.

Cardiac I/R injury in diabetes is associated with overproduction of ROS, Ca^2+^ overload, mitochondrial damage, inflammation, and so on. Necroptosis plays a central role in diabetic cardiomyopathy and cardiac I/R injury through a complex interplay with these harmful events. During cardiac I/R injury, ROS not only activates necroptosis via RIPK3/MLKL signaling but also is further amplified by necroptosis itself, establishing a vicious cycle and further amplifying oxidative stress and worsening cardiac injury (17, 52). Necroptosis is also closely associated with inflammation during cardiac I/R. TNF-α and continuous activation of the death receptor pathway promote RIPK3/MLKL-related necroptosis, exacerbating inflammatory cascade reactions and cardiomyocyte death (17, 19, 53). In addition, it has been highlighted that mPTP opening, a pivotal constituent in the execution of necroptosis, causes rapid mitochondrial swelling, loss of ΔΨm, and mitochondrial respiratory dysfunction, leading to overproduction of mitochondrial ROS and ultimately resulting in necroptosis (17, 54). Importantly, activated RIPK3 can be transported to the mitochondrial membrane continuously triggering mPTP opening through multiple pathways, thus establishing a positive feedback loop and hastening necroptosis in diabetic cardiomyopathy and I/R heart (17, 55). These phenomena or results suggest that RIP3/MLKL-mediated necroptosis connects oxidative stress, inflammation, and mPTP opening, which jointly determine the high prevalence and poor prognosis of post-resuscitation injury in diabetic hearts. Our results confirm that luteolin inhibits RIPK3/MLKL activation, reduces ROS and inflammatory cytokines, and preserves mitochondrial function, suggesting that suppression of necroptosis is a critical component of its cardioprotective action. Moreover, the present study also shows that the increased cytosolic free Ca^2+^, mPTP opening, disruption of ΔΨm, dysfunction of mitochondrial respiratory chain complexes, and decreased ATP production in diabetic hearts after resuscitation are all significantly reversed by luteolin, suggesting that preserving mitochondrial integrity by luteolin benefits in suppressing necroptosis and protecting the diabetic against post-resuscitation injury. However, the mechanism by which luteolin improves mitochondrial function and inhibits necroptosis in the diabetic heart after CA/CPR needs further investigation.

Sirt3, a mitochondrial deacetylase, is known to preserve mitochondrial function and suppress necroptosis in diabetic cardiomyopathy (22, 24) and reduce ROS via activation of SOD2 in cardiac I/R injury (21, 25, 26). Recently, Sirt3 has been reported to ameliorate post-resuscitation myocardial dysfunction (28). These results suggest that up-regulation of Sirt3 may exert potent protective effects against post-resuscitation injury in diabetic hearts. It is worth noting that luteolin effectively inhibits ROS generation and improves mitochondrial function through up-regulating Sirt3 in the cerebral tissues exposed to I/R injury and in ultraviolet radiation B-damaged skin (30, 31), indicating that Sirt3 may be the target of luteolin in attenuating diabetic post-resuscitation myocardial dysfunction. The present study demonstrates that luteolin significantly activates Sirt3 and SOD2 in diabetic rat hearts following CA/CPR, and that its cardioprotective effects are abolished by the Sirt3 inhibitor 3-TYP. Furthermore, the diminishing effects of luteolin on ROS production, inflammatory response, cytosolic Ca^2+^ overload, mPTP opening, loss of ΔΨm, disruption of mitochondrial oxidative phosphorylation, and activation of necroptosis-related factors are all halted by 3-TYP. Convergently, it is feasible that luteolin targets Sirt3 to suppress necroptosis and post-resuscitation injury in diabetic hearts. While luteolin was recently demonstrated to enhance Sirt3 expression through miR-125b-5p inhibition in osteoporotic rat bone tissue (56), suggesting that this miRNA-mediated mechanism may similarly up-regulate Sirt3 signaling in the post-resuscitation diabetic heart. However, the precise pathway through which luteolin directly or indirectly modulates Sirt3 to alleviate resuscitation injury in diabetic hearts remains to be fully elucidated.

In summary, luteolin confers cardioprotection against post-resuscitation injury in diabetes primarily by activating the Sirt3 signaling pathway. Sirt3 reduces oxidative stress through SOD2 activation, thereby limiting inflammatory responses and preserving mitochondrial function. These synergistic effects of Sirt3, enhanced by luteolin, suppress necroptosis and protect diabetic hearts against post-resuscitation injury. Although our results indicate luteolin’s cardioprotective potential in diabetic CA/CPR models through Sirt3-mediated pathways, its clinical translation requires systematic evaluation of pharmacokinetics, long-term safety profiles, and clinical trials before therapeutic application can be considered.

## Data Availability

The original contributions presented in this study are included in this article/supplementary material, further inquiries can be directed to the corresponding author.

## Footnotes

1
http://diabetesatlas.org/atlas/tenth-edition/
